# Marble Powder as a Soil Stabilizer: An Experimental Investigation of the Geotechnical Properties and Unconfined Compressive Strength Analysis

**DOI:** 10.3390/ma17051208

**Published:** 2024-03-05

**Authors:** Ibrahim Haruna Umar, Hang Lin

**Affiliations:** School of Resources and Safety Engineering, Central South University, Changsha 410083, China; ibrahimharunaumar@yahoo.com

**Keywords:** soil stabilization, marble powder, unconfined compressive strength (UCS), fine-grained soil, exploratory data analysis (EDA), geotechnical properties

## Abstract

Fine-grained soils present engineering challenges. Stabilization with marble powder has shown promise for improving engineering properties. Understanding the temporal evolution of Unconfined Compressive Strength (UCS) and geotechnical properties in stabilized soils could aid strength assessment. This study investigates the stabilization of fine-grained clayey soils using waste marble powder as an alternative binder. Laboratory experiments were conducted to evaluate the geotechnical properties of soil–marble powder mixtures, including Atterberg’s limits, compaction characteristics, California Bearing Ratio (CBR), Indirect Tensile Strength (ITS), and Unconfined Compressive Strength (UCS). The effects of various factors, such as curing time, molding water content, and composition ratios, on UCS, were analyzed using Exploratory Data Analysis (EDA) techniques, including histograms, box plots, and statistical modeling. The results show that the CBR increased from 10.43 to 22.94% for unsoaked and 4.68 to 12.46% for soaked conditions with 60% marble powder, ITS rose from 100 to 208 kN/m^2^ with 60–75% marble powder, and UCS rose from 170 to 661 kN/m^2^ after 28 days of curing, molding water content (optimum at 22.5%), and composition ratios (optimum at 60% marble powder). Complex modeling yielded R^2^ (0.954) and RMSE (29.82 kN/m^2^) between predicted and experimental values. This study demonstrates the potential of utilizing waste marble powder as a sustainable and cost-effective binder for soil stabilization, transforming weak soils into viable construction materials.

## 1. Introduction

Weak or poor soil conditions pose significant challenges in construction projects, often leading to stability issues, settlement, and bearing capacity problems [1]. Soil stabilization is a technique used to improve the engineering properties of soils, such as strength, stiffness, permeability, and durability [1,2]. It is often applied to fine-grained soils prone to swelling, shrinkage, compaction, and liquefaction [3]. Among the various stabilizers used for soil improvement, marble powder is a promising material that can be obtained from the waste generated by the marble industry [4]. Marble powder is composed of calcium carbonate, which can react with clay minerals and cement the soil particles. Marble powder improves soil properties through pozzolanic, cation exchange and carbonation reactions that cement particles, modify plasticity, and neutralize deleterious alkalis over time. The calcium ions released play a key role in inducing these beneficial changes that increase stability [5]. In addition, marble powder is an environmentally friendly and cost-effective alternative to conventional stabilizers, as it can reduce the disposal of industrial waste and the consumption of natural resources [1,6,7].

Soil stabilization is crucial in enhancing soil properties for construction and other applications [8,9,10,11,12,13,14,15,16,17]. Laboratory experiments on soil–marble powder mixtures provide insights into their geotechnical properties, including Atterberg’s limits, compaction characteristics, CBR, ITS, and UCS [18]. Marble powder can improve soil’s geotechnical parameters, reducing plasticity and swelling and increasing UCS. This suggests its potential as a soil stabilizer, especially for soils with high clay content. Factors such as curing time, molding water content, and composition ratios significantly impact UCS [1]. For instance, UCS increases with clay content and cement–tailing ratio but decreases with molding moisture content. These findings are vital in understanding soil–marble powder mixtures’ behavior under different conditions, aiding in informed decisions in geotechnical engineering and construction [19]. Thus, soil stabilization using marble powder can significantly enhance the soil’s geotechnical properties, making it more suitable for construction and other applications. The choice of stabilization method and the quantity of marble powder to be added can be guided by the Atterberg limits and other soil properties [20].

Several studies have investigated waste marble powder as a stabilizer for fine-grained soils. These studies have generally found that marble powder can effectively strengthen clayey subgrade and increase its bearing capacity for traffic loads [1,5,8,21,22,23,24,25,26,27,28,29,30]. For example, Sivrikaya et al. investigated the efficiency of marble powder in stabilizing fine-grained soils regarding volume change. They found that marble powder reduced the plasticity, expansion, and shrinkage of soils and increased the UCS, especially at 50% marble powder content [31]. Umar et al. analyzed clay soil stabilization using marble powder and developed Artificial Neural Network (ANN) models to predict stabilized soils’ UCS and Ultrasonic Pulse Velocity (UPV). They reported that the ANN models accurately predicted the UCS and UPV values with high correlation coefficients [1]. However, there is limited research on the comprehensive evaluation of the geotechnical properties and long-term performance of soil–marble powder composites, particularly for fine-grained clayey soils. In addition, the literature lacks a comprehensive and reliable predictive model that can estimate the UCS of marble-powder-stabilized soils based on a simple and robust mathematical function, such as a quartic (fourth-degree) polynomial regression.

This study attempts to fill this gap in the literature by developing a novel quartic polynomial regression model to predict the UCS of fine-grained soils stabilized with marble powder. Unlike previous studies, this research utilizes a comprehensive laboratory experimental program that includes different types of fine-grained soils and marble powder and different marble powder proportions, water contents, and curing times. The study also uses statistical analysis to establish relationships between the UCS and the influencing factors and evaluate the predictive model’s accuracy and validity. The study aims to provide insight into the viability of recyclable marble powder for soil stabilization and to offer a simple and effective approach for performance prediction and infrastructure implementation. It also investigates the feasibility of using waste marble powder as an alternative binder for stabilizing fine-grained clayey soils, addressing the need for eco-friendly and cost-effective construction materials.

This study investigates waste marble powder’s potential as a sustainable binder for stabilizing fine-grained clayey soils. This study stabilizes a specific fine-grained clayey soil sample obtained from Jingdezhen City, Jiangxi Province, China, using marble powder from a single industrial source in Yunfu, Guangdong Province, China. The research is limited to laboratory-scale experiments and does not address field implementation or long-term performance under actual environmental conditions. Laboratory experiments were conducted to characterize the geotechnical properties of soil–marble powder mixtures, including Atterberg’s limits, compaction, CBR, ITS, and UCS tests, following relevant ASTM standards. A range of marble powder contents (0–75% by dry weight) and curing times (0–28 days) were investigated to assess the effects of composition and curing duration on soil properties. Exploratory Data Analysis (EDA) techniques, such as histograms, box plots, and statistical modeling, were employed to analyze the influence of various factors on UCS and identify optimal conditions for soil stabilization.

## 2. Materials and Method

### 2.1. Materials

**Figure 1:**
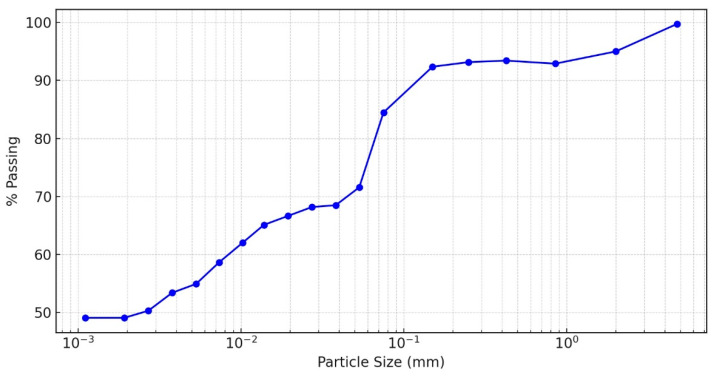
Particle size distribution curve.

The natural soil samples used in this study were obtained from Jingdezhen City, Jiangxi Province, China. Based on the Unified Soil Classification System standards, basic geotechnical classification tests categorized these soils as high-plasticity clays (CH). Particle size distribution analysis using sieve and hydrometer tests revealed a fine-grained soil composition, with over 70% of particles passing the 200-mesh sieve, as shown in the log scale gradation curve (Figure 1).

Soil and marble powder samples were oven-dried at 105 ± 5 °C prior to mixing to standardize moisture content. Compacting soil mixtures prepared cylindrical specimens, 38 mm in diameter and 76 mm in height and 0–75% marble powder by total dry weight using a wet tamping compaction method according to established standards. A three-layer compaction method was used to improve density uniformity. The samples were then cured and sealed in plastic at controlled moisture levels of 20%, 22.5%, or 24.5% dry weight. Curing was performed for 0, 3, 7, 14, or 28 days in humidity chambers maintained at 20–28 °C to maintain the target moisture content throughout the specified curing period. Unconfined compressive strength (UCS) test was performed after special sample preparation. UCS evaluations for evaluating axial load capacity followed standardized method (ASTM D2166) [32]. Careful control of parameters such as dimensions, compaction energy, curing conditions, testing machine calibration, and data acquisition enabled reliable characterization of strength trends across formulations and curing duration. Finally, after being extruded from the molds, the samples were immediately re-wrapped in plastic membranes and cured in humidity-controlled cabinets to retain the target moisture levels throughout the designated curing durations of up to 28 days. The matrix of compositional ratios, moisture contents, and curing days provided multidimensional performance data according to the outlined test program (Table 1) and Table 2 contain basic soil properties [1].

Specific gravities around 2.7 are typical of soils dominated by silicate minerals such as quartz, feldspars, micas, chlorite, kaolinite, etc. [33]. This is consistent with the identification of kaolinite as the dominant mineral. Silicates have densities in the range of 2.6 to 2.8 [34]. If the soil contained abundant iron oxides or iron minerals such as hematite, the specific gravity would be higher, around 3.5 or more. Iron has a density above 5 [35,36]. The dark beige description also suggests the absence of significant iron, as iron oxides would likely give the soil an orange/red/brown color. Therefore, based on the evidence, this soil is composed primarily of silicate minerals, such as kaolinite, with no significant iron content. The specific gravity indicates a typical silicate-based particle density with no iron enrichment. The absence of iron is consistent with the observed soil color.

The marble powder used in this experiment was sourced from Yunfu, a city in Guangdong Province, China. The marble powder was derived from the city’s industrial waste, specifically from the manufacturing industry. Table 3 comprehensively lists the minerals and compounds found in marble powder [1].

### 2.2. Methods

**Figure 2:**
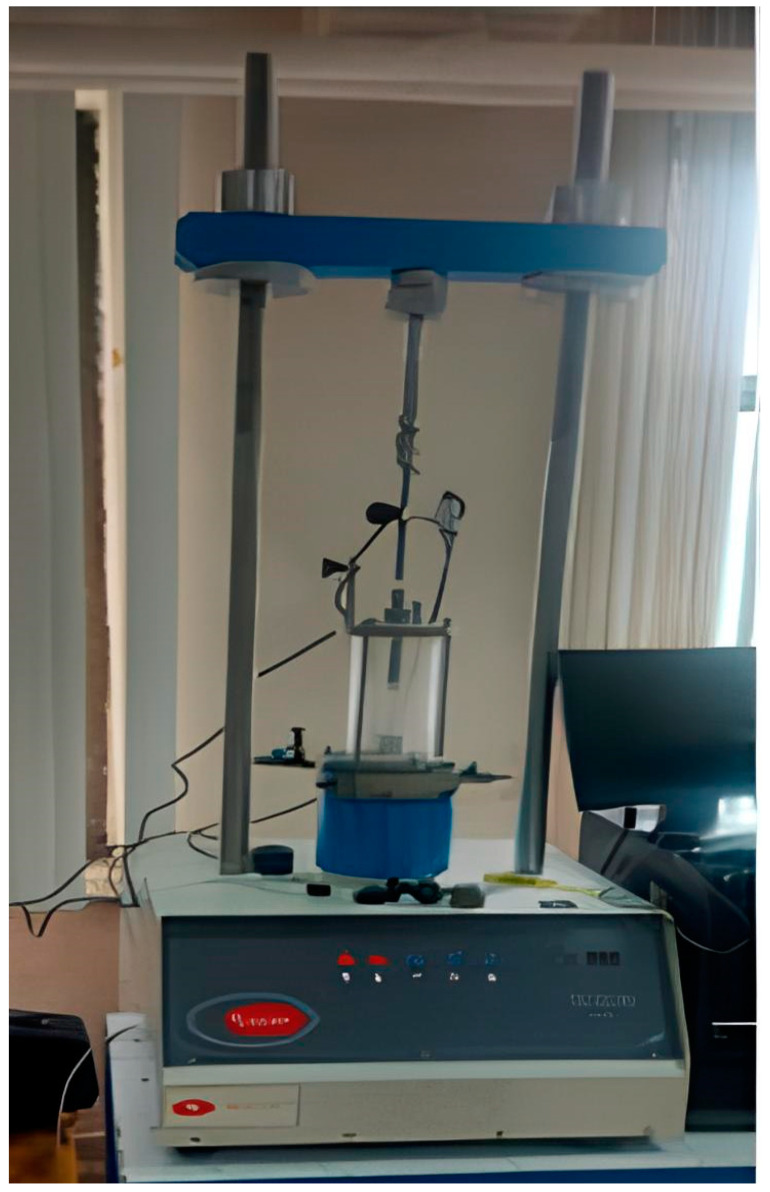
UCS machine.

The Unconfined Compressive Strength (UCS) test was conducted per the ASTM D2166 standard to quantify the compressive resistance of natural and stabilized soil specimens [37]. Cylindrical samples were placed in the UCS apparatus without constraints and axially loaded at a constant strain rate of 0.076% per minute. Continuous measurement of the resulting load and deformation was performed until failure by sudden fracture of the sample, as illustrated in Figure 2. The load–deformation profiles were analyzed to identify the ultimate axial load sustained before rupture. The maximum compressive stress, UCS, was obtained by dividing this peak load by the original cross-sectional area. Careful determination of parameters, including sample dimensions, curing methods, loading rates, and data acquisition, enabled accurate, standardized measurement of soil strengths for comparison across compositions and time points. The simplified, unconfined testing allows for ready insight into the improvements induced by the marble powder stabilization. Replicating the experimental methodology facilitates equitable comparison and systematic optimization in future studies.

### 2.3. Exploratory Data Analysis (EDA)

Exploratory Data Analysis (EDA) encompasses a range of quantitative and visual techniques to comprehensively summarize and investigate key characteristics within datasets without formal statistical modeling or hypotheses [38]. By graphing distributions, relationships, anomalies, etc., EDA reveals preliminary insights to guide analysis choices [39]. Primarily, EDA enables a free-form understanding of what stories data can tell beyond constrained inference testing or assumptions [40]. It facilitates discovering patterns, quantifying variability, and visually screening for errors, outliers, etc. This data-first approach checks the validity of championed analysis methods against the complexity revealed, improving selection [41]. Overall, EDA forms an integral upfront checkpoint that enhances any subsequent sophisticated analysis by grounding choices in robust feature awareness rather than blind application. Enhanced realization of subtle nuances such as confidence bounds, latent categories, skew, etc., also prepares data for advanced analytics such as machine learning [42]. Both uni- and multivariate visual and mathematical EDA thoroughly profiling dataset properties are crucial first steps that widely influence overall analysis quality. The flexible revelations ensure analyses answer the right questions [43].

#### Histogram and Boxplot

**Figure 3:**
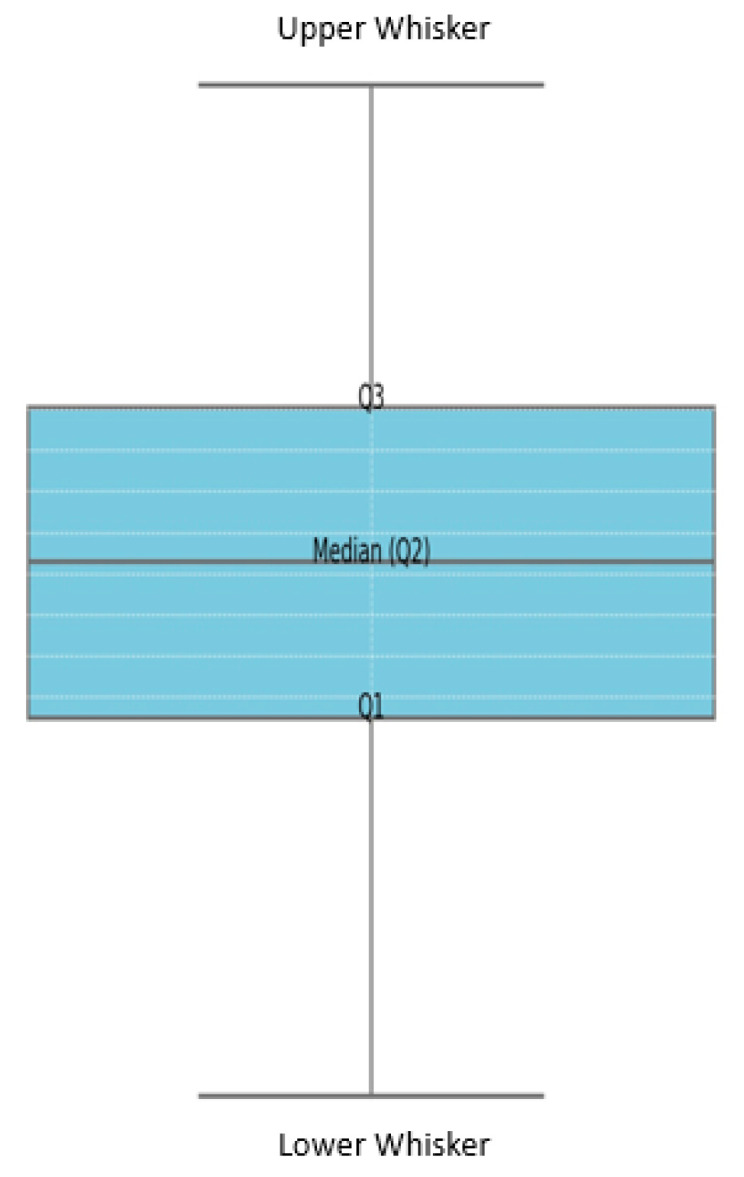
Component of boxplot.

Histograms and box plots are widely used graphical techniques for visualizing statistical distributions. Histograms plot binned data frequencies on y-axes against user-defined ranges along x-axes, conveying aggregation patterns. Box plots compactly represent the spread and skewness of numerical data using five summary statistics—minimum, first quartile, median, third quartile, and maximum. The central box spans the interquartile range, whiskers indicate value dispersion, and outliers are isolated points. Because of this complementary utility for easily visualizing and interpreting numerical data characteristics, histograms and box plots find ubiquitous application across analytical domains, including common paired displays for exploratory analysis. While granular details of the stylistic components and comparative advantages have been extensively noted in the prior statistics literature, their core purpose is the concise graphical translation of distributional insights. For the current research context, augmenting the presented analytical results with these established visualization tools would provide intuitive visual support for rapid analysis by readers. However, elaborating on the well-documented usage conventions in the main text may not enhance the key chemical and mechanical insights conveyed. Therefore, retaining the visualizations while condensing the descriptive background details as a generally known methodology would optimize the length and clarity of the paper. Figure 3 shows the component of the boxplot.

### 2.4. Statistical Modeling

The statistical modeling involved fitting a linear regression model to understand the relationship between UCS and other variables, such as curing time, molding water content, and composition ratios.

#### 2.4.1. Quartic Polynomial Regression Model

A quartic (fourth-degree) polynomial regression model is a type of polynomial regression that involves a fourth-degree polynomial function. Quartic regression fits a quartic function to a set of data points. Quartic regression fits a curve to a set of data points [43]. The equation becomes quite extensive due to the inclusion of all possible combinations of these variables up to the fourth degree and has the following form:

$$UCS=\beta_{0}+\beta_{1}x_{i}+\beta_{2}w+\beta_{3}x_{s}+\beta_{4}x_{p}+\beta_{5}x_{i}^{2}+\beta_{6}w^{2}+\beta_{7}x_{s}^{2}+\beta_{8}x_{p}^{2}+\ldots +\beta_{13}x_{i}^{4}+\beta_{14}w^{4}+\beta_{15}x_{s}^{4}+\beta_{16}x_{p}^{4}+\;[\text{Interaction Terms up to the fourth degree}]+\varepsilon$$

where

UCS=unconfined compressive strength
Coefficient (β) = each variable characteristics on the effect of UCS
$\beta_{0}=\text{value of UCS when all the independent variables are zero}$
$x_{i}=$ curing time
w= molding water content
$x_{s}=$ soil ratio
$x_{p}=$ powder ratio
Errorterm(ε) = difference between the observed and predicted values of UCS

#### 2.4.2. Model Evaluation

Rigorous validation of model accuracy in linking mechanisms to infrastructure performance is imperative. The Root Mean Squared Error (*RMSE*) metric is an essential statistical tool for evaluating model fidelity.

The performance of the regression model is evaluated using the Root Mean Squared Error (*RMSE*).

$$RMSE=\sqrt{\frac{1}{n}\sum_{i=1}^{n}(y_{i}-\hat{y}{)}^{2}}$$

where

n= number of samples
$y_{i}=$ actual value of the response variable
$\hat{y}=$ predicted value of the response variable

The Coefficient of Correlation (R) value measures the linear relationship between the estimations and the actual values. The R-value is calculated using the following formula:

$$R=\frac{n(\sum y.y_{p})-(\sum y)(\sum y_{p})}{\sqrt{[n\sum y^{2}-(\sum y{)}^{2}][n\sum y_{p}^{2}-(\sum y_{p}{)}^{2}]}}$$

Mean of the observed data = $ỹ=\frac{1}{n}\sum (y_{i})$
The total sum of squares = $\sum_{i=1}^{n}(y_{i}-ỹ{)}^{2}$
Residual sum of square = $\sum_{i=1}^{n}(y_{i}-y_{p}{)}^{2}$
Coefficient of determination R^2^ = $1-\frac{Total\;sum\;of\;residual}{Total\;sum\;of\;sqaure}$

where y and y_p_ are the actual and the predicted values; ỹ and ỹ_p_ are the average of the actual and the predicted values, respectively; n is the sample size.

## 3. Results and Discussion

### 3.1. Atterberg’s Limits Characteristics

**Figure 4:**
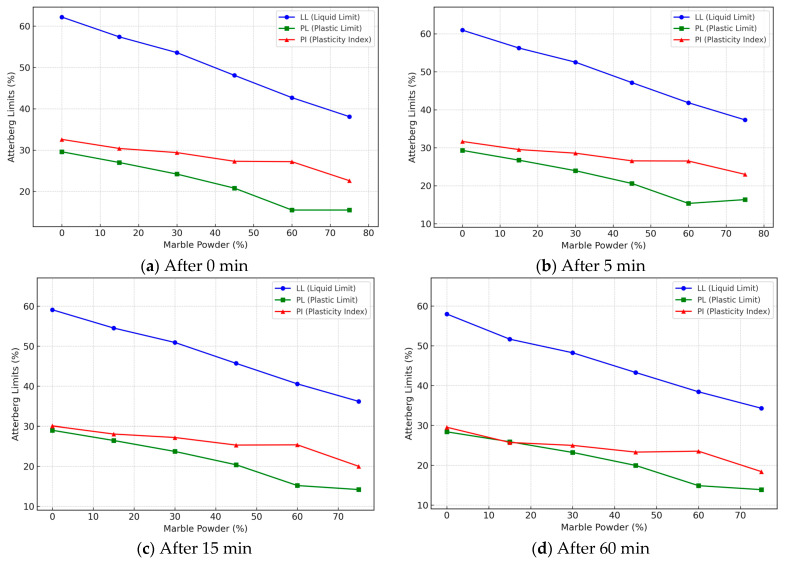
Atterberg’s limits.

Figure 4 shows how marble powder admixture affects natural soil’s Atterberg’s limits (Liquid Limit, Plastic Limit, and Plasticity Index) at different percentages (0%, 15%, 30%, 45%, 60%, 75%) of marble contents after 0, 5, 15, 60 min, conducted by ASTM D4318 [1]. The results indicate that as the marble powder content increases, the Liquid Limit (LL), Plastic Limit (PL), and Plasticity Index (PI) decrease over time. However, the reduction is marginal over time but more pronounced at 60 min.

#### 3.1.1. Effect of Marble Powder Incorporation on Liquid Limit (LL) over Time

The LL decreases from 62.2% for 100% natural soil to 38.1% for 75% marble powder after 0 min. After the 5-min test, adding more marble powder decreased LL plasticity from 60.956% to 37.338%. The 15-min test indicates a reduction in LL from 59.09% to 36.195% at 75% marble powder. The findings of the 60-min test indicate that LL decreased from 57.98% to 34.29% for 75% marble powder. This aligns with findings by Wathiq et al., who reported a decrease in LL from 51.2 to 36.1 with Cement and By-Product Petrit T addition after a mellowing period [44]. The reduction in LL over 15 and 60-min times is also consistent with Prasad et al., who showed an 8% decrease in LL over a 48-h time with 10–20% lime admixtures. Moreover, Bayat et al. reported a decline in LL with cement and lime addition after 30 min [45]. The reduction is caused by mechanisms involving cation exchange and flocculation–agglomeration effects [1].

#### 3.1.2. Effect of Marble Powder Incorporation on Plastic Limit (PL) over Time

As the percentage of marble powder increases after 0 min, the PL decreases from 29.6% to 15.5%, indicating that less water is required for plasticity. The addition of marble powder decreases PL from 29.304% to 16.345% after 5 min at 60% powder. The 15-min test indicates a reduction in PL from 29.008% to 14.19% at 75% marble powder. The findings of the 60-min test indicate that PL decreased from 28.416% to 13.88% for 75% marble powder. This aligns with literature that states PL changes little after an initial adjustment period of some stabilizers, such as Rice Husk Ash (RHA), for instance, research by Brooks, with 25% RHA decreasing PL by 46% while only reducing LL by 13%. The significant sensitivity of PL is due to the modification of the clay fraction and the dominance of frictional behavior, as noted by Kumar et al. [46]. The decrease in PL over time is like the findings of Kazemian et al., who reported reductions of over 10% after 15–30 min for soils treated with 10% nano-CuO. Thus, an ongoing physicochemical interaction is indicated [44].

#### 3.1.3. Effect of Marble Powder Incorporation on Plasticity Index (PI) over Time

The PI remains between 27 and 32% for marble powder percentages between 0 and 60% but decreases to 22.6% at 75% marble powder after 0 min. PI remains at 26–30% for 0–60% marble powder but drops to 22.993% at 75% powder contents after 5 min. The 15-min test indicates a decrease in PI from 25 to 30% to 20.005% at 75% marble powder. The findings of the 60-min test indicate that PI remained stable at 23–29% before declining to 18.41% for 75% marble powder. A slower rate was observed from 15 to 60 min. Such reduction in plasticity suggests that physicochemical effects occur during prolonged conditions. This time-dependent behavior is like lime-cement-treated soils, where most PI reduction occurs early [45]. The overall PI trend supports the research conducted by Pourakbar and Asadi on 45% alkali-activated agro-waste mixes. However, its values were more variable over 72 h due to flocculation–deflocculation effects [47].

In another paper by Sampathirao et al., the effects of lime, gypsum, and quarry dust additions were investigated. They found that adding up to 25% gypsum reduced the Plasticity Index (PI) from 21.4 to 16.7%. This reduction was attributed to the cation exchange affects that decreased the thickness of the diffuse double layer [48]. Cokca assessed soils treated with Cement Kiln Dust (CKD), reporting a significant decrease in Plasticity Index (PI) from 38.7% to 18.2% with 10% CKD content after 7 days. This decrease was attributed to the elevated pH, which induced flocculation and cementitious reactions over time. In the current study, adding marble powder resulted in a comparatively lower reduction in PI, from 27.2% to 22.6%, with a 60% addition after 1 hour. Its improved workability highlights the potential specialty advantages of marble powder, but lower reactivity compared to conventional stabilizers such as lime and CKD. Particle packing and filler effects appear more dominant than chemical interactions [49].

### 3.2. Modified Proctor Compaction Characteristics

**Figure 5:**
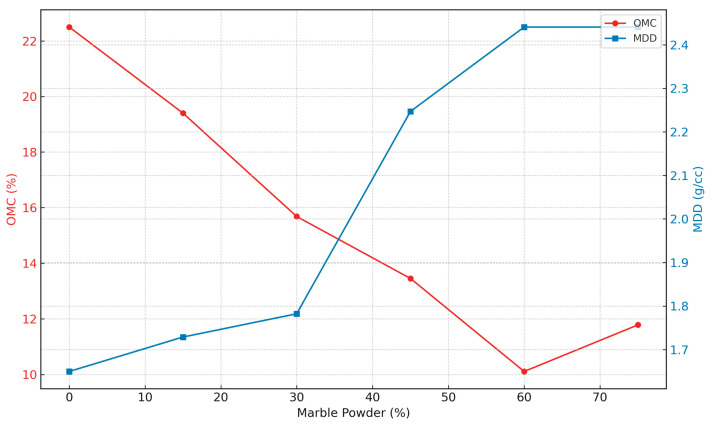
Compaction characteristics.

Figure 5 shows the results of modified Proctor compaction tests conducted by ASTM D1557 on natural soils substituted with marble powder at increments by dry weight, ranging from 0% to 75%. Maximum Dry Density (MDD) results show an increasing trend as the marble powder content increases. The MDD increases from 1.65 g/cc for the neat soil to 2.441 g/cc at 60 and 75% marble powder. This is consistent with Sabat’s findings, which reported up to 32% higher MDD increase for quarry dust-amended soils. The increment in MDD reflects the densification of the soil structure due to agglomeration into coarser fractions [50]. However, the increase in the rate of MDD becomes marginal beyond 45% marble powder. This may indicate a threshold replacement level before which significant density changes manifest. This non-linearity is like the patterns documented for soils modified with fly ash [51]. Contrastingly, OMC exhibits a steady decrease from 22.5% for neat soil to 10.11% for the 60% blend. This lower moisture demand is consistent with previous studies on lime-treated soils [52] and is likely due to the reduction surface area available for water adsorption. Additionally, the nearly proportional increase in MDD with increasing marble powder dosage is consistent with findings for sand–bentonite mixtures amended with marble waste [53]. This suggests that the intrinsic water retention capacity may predominantly govern changes in moisture content.

### 3.3. California Bearing Ratio (CBR) Characteristics

**Figure 6:**
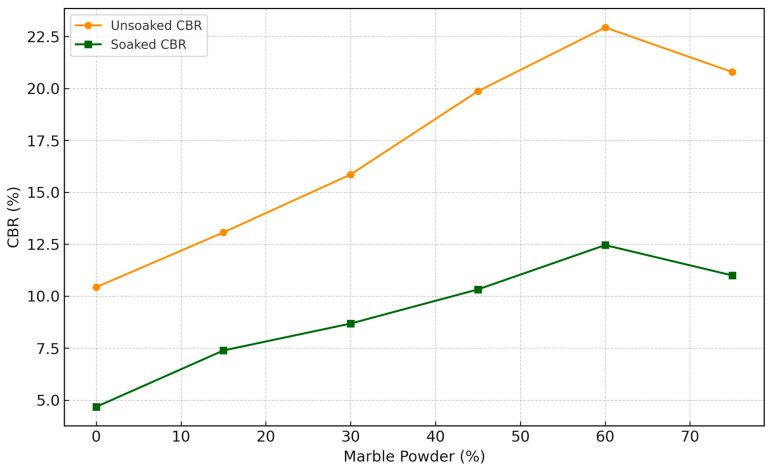
California Bearing Ratio (CBR).

The California Bearing Ratio (CBR) is a crucial parameter in geotechnical engineering, as it indicates the load-bearing capacity of soils, which is essential for designing road subgrades, pavements, and embankments. The results of this study show a clear correlation between the marble powder content and the CBR values of the modified soils. As presented in Figure 6, the results of the CBR test on natural soil samples blended with varying marble powder content from 0% to 75%, as performed by ASTM D1883 [54].

#### 3.3.1. Effect of Marble Powder Incorporation on Unsoaked CBR

The unsoaked CBR increased progressively from 10.43% for the neat soil to a peak of 22.94% at 60% marble powder, signifying an improvement in the modified soils’ strength and bearing capacity properties. This aligns with similar improvement trends reported for soils treated with lime [51] and marble dust [55]. However, a marginal drop is noticeable beyond 60%, with the CBR reducing to 20.79% at 75% marble powder. This indicates a potential optimum replacement level, corroborating observations made by Kolias et al. for cement kiln dust-amended clayey soils, which depicted peaks before declining [50].

#### 3.3.2. Effect of Marble Powder Incorporation on Soaked CBR

While the soaked CBR values are anticipatedly lower than unsoaked CBR, they follow a comparable increasing relationship from 4.68% to 12.46% with marble powder dose up to 60% due to moisture resistance afforded by enhanced interparticle bonding. However, the decrease in strength is more significant when the marble powder content exceeds 60%, as observed in the unsoaked CBR. This susceptibility to moisture at high replacement ratios is consistent with findings on soils modified with quarry dust [51].

### 3.4. Indirect Tensile Strength (ITS) Characteristics

**Figure 7:**
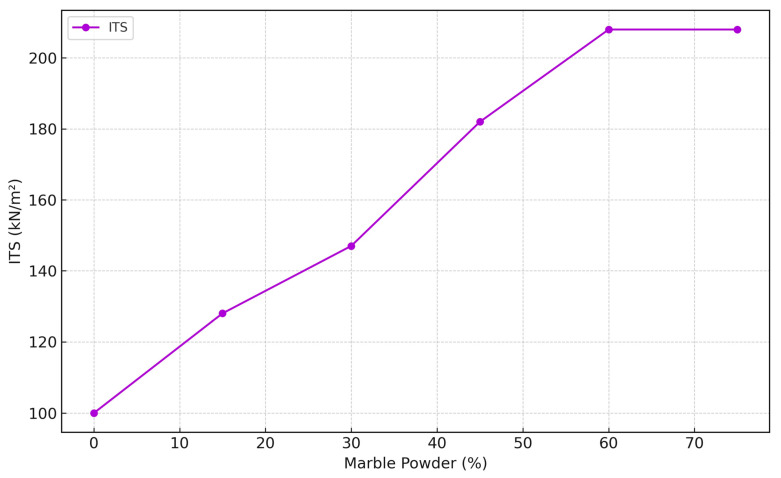
Indirect Tensile Strength (ITS).

Indirect Tensile Strength (ITS) measures a material’s tensile strength, which is the resistance to being pulled apart. In geotechnical engineering, ITS is often used to assess the tensile strength of materials such as soil and rock. ITS is an important parameter in designing projects since the tensile strength of earth materials is significantly lower than their compressive strength. Understanding the ITS of a material can help engineers predict how it will behave under tensile stress, which is crucial when designing structures such as tunnels, bridges, or dams that may be subjected to these types of forces. Moreover, the ITS test can be significantly higher than the direct tensile strength test value. Therefore, it provides a conservative estimate of the material’s strength, ensuring safety in design and construction. As presented in Figure 7, the results of ITS test on natural soils blended with marble powder from 0% to 75%, as performed by ASTM D4644 [56]. The results showed a progressive increase in ITS from 100 kN/m^2^ for neat soil to a peak of 208 kN/m^2^ at both 60% and 75% marble powder content. Marble powder stabilization significantly improves tensile capacity, like the improvements seen in cement-modified soils [55]. However, the incremental rate of improvement decreases beyond 30% marble powder and plateaus after 60% replacement. This trend is consistent with observations made by Kolias et al., where the tensile strength of lime-treated clays peaked at a certain stabilizer dose, indicating an optimum amendment level [50].

The ITS tests demonstrate the potential of marble powder as a soil stabilizer. However, careful consideration must be given to the amount of marble powder used, as excessive amounts may not yield additional benefits and could potentially degrade the performance of the soil. Future research should focus on determining the optimum marble powder content for diverse types of soils and under various environmental conditions. This will help to maximize the benefits of marble powder stabilization and promote its wider application in geotechnical engineering.

### 3.5. Unconfined Compressive Strength (UCS) Characteristics

Based on the data in Table 4, the Unconfined Compressive Strength (UCS) generally increases from 0 to 28 days of curing for all soil/powder compositions and molding water contents. This indicates the expected increase in strength as the specimen cures over time. The increase in strength over extended curing periods is consistent with expectations for stabilizing materials such as cement or lime. Ongoing hydration of the cementitious constituents produces additional binder products over time. The literature also discusses the influence of factors such as curing time and moisture content. Comparing the rate of increase found here to established models may differentiate the behavior of this powder from conventional cement [1].

At each level of molding water content, the UCS values initially tend to increase as more powder is added to the soil mix, up to about 40–60% powder content. However, at 75% powder, the strength decreases compared to lower powder percentages. This suggests that there is an optimum powder content for maximum strength gain. While additive powders such as fly ash often improve strength, the decrease at 75% of powder differs from the peak or plateau usually observed. This suggests that unique reactions may occur with high amounts of this powder in the soil mix. Supplemental chemical analysis could help determine if there are decreasing reactants from the soil, excessive water requirements, or other negative interactions with high powder content [1]. The highest UCS values were obtained at the optimum molding water content of 22.5% at each curing time. The 24.5% water content consistently produced the lowest UCS values. This indicates that excessive water reduces the strength gain during curing. The reduction in compressive strength with increasing water content follows the established principles of water–cement ratio effects known for concrete. However, conventional Portland cement mixes rarely reach water content as high as 24.5%. The sustained strength gain here indicates that this powder can support higher moisture levels than conventional cementitious stabilizers such as lime before severe strength loss occurs.

Table 5 statistical data show the progression of Unconfined Compressive Strength (UCS) development for three water contents—20%, 22.5%, and 24.5%—as a function of increasing cement curing age from 0 to 28 days. An ideal replication would include five replicates per sample group. This would provide robust UCS averages along with solid statistical assessments of variation. Differences between water content and curing time could also be more clearly distinguished. Across all molding conditions, there is initially negligible strength with a mean 0-day UCS of 237 to 352 kN/m^2^. This is expected prior to significant cementitious reactions in the unstabilized composite. However, clear time-dependent gains are manifested over the subsequent day of curing, consistent with the accumulation of cementitious gel residues that bind the soil matrices. Optimal UCS enhancement occurs at the intermediate moisture level of 22.5%, with mean 28-day values of 546 kN/m^2^ compared to 378 and 443 kN/m^2^ for the 20% and 24.5% conditions, respectively. This is consistent with the narrowed 95% confidence intervals, indicating less variability and outliers. Excessive or insufficient moisture availability will likely inhibit cement hydration and pozzolanic activity. The standard deviation values increase progressively with curing time, from 45 to 152 kN/m^2^ for the 28-day 22.5% series. This suggests that the variability in the complex precipitation bond increases with curing time. However, even the lower 95% CI limit of 386 kN/m^2^ at 28 days indicates high strength reliability.

The observed immature 0-day strength followed by a 5-fold increase in compressive strength over the 28-day curing period is consistent with previous findings of progressive improvements in bonding and matrix stability in pozzolana-treated soils over time [1]. The quantitative magnitude and temporal rate are also consistent with findings for cement-stabilized clay soils [4]. However, the revelation of an elevated 22.5% moisture content that consistently maximizes UCS development across the maturation period contrasts with Petry et al.’s indication of invariant strength gains above 20% moisture. The expansive 95% confidence intervals may obscure such intermediate optima, even for peak formulations in said study. Elucidation of a narrow mold water content window centered at 22.5% that enhances and stabilizes the long-term strength maturation of soil-cement composites introduces the potential for significant improvement over current practices, assuming invariant performance above 20% moisture. Recognition of this optimized moisture criterion preserves the enhanced cementitious reactivity while avoiding the well-established strength losses above 25% levels. The sensitivity highlights that cement-soil reactions are as dependent on controlled moisture ratios as on binder fractions. Optimized moisture tuning coupled with sufficient aging can achieve unprecedentedly durable infrastructure materials from marginal soils through intentional stabilization. The methodology demonstrates that even precipitation mechanisms thought to be well established still contain overlooked nuances that affect quantitative results.

### 3.6. Distribution of UCS Values

**Figure 8:**
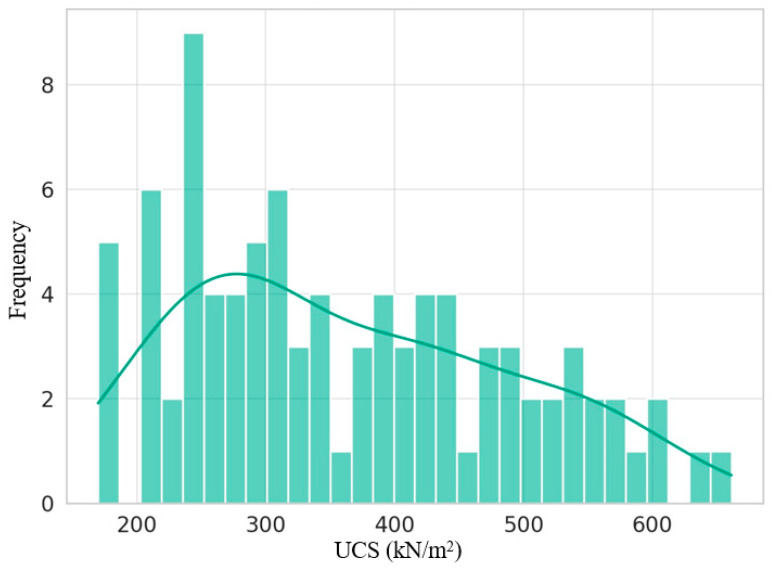
Distribution of Unconfined Compressive Strength (UCS) values for the fine-grained soil samples stabilized with marble powder.

Figure 8 indicates a slightly right-skewed distribution of Unconfined Compressive Strength (UCS) values for the fine-grained soil samples stabilized with marble powder. This shows that while most of the UCS values are concentrated in the center of the distribution (around 170–661 kN/m^2^), some higher outlier values extend the distribution’s right tail. The concentration of UCS values in the central tendency indicates that adding marble powder resulted in an overall increase in strength for most of the stabilized soil samples. However, the right skew shows some variability in the effectiveness of the stabilization, with a few samples showing exceptionally high gains in UCS compared to the average. A right-skewed distribution often indicates that additional factors, variables, or issues related to sampling or testing procedures may contribute to the high outlier values. For this stabilized soil, variations in marble powder content, differences in soil mineralogy or grain size distribution, inconsistencies in sample preparation, curing methods, or the UCS test itself may be contributing factors.

Using waste marble powder for soil stabilization can recycle waste materials, reduce the mining and production of traditional cementitious binders, and provide a sustainable and cost-effective construction material. The right-skewed distribution highlights the ability of this technique to achieve very high increases in strength under certain conditions. Further refinement could optimize the factors controlling the outlier high values to develop soil–marble composites with both consistent and exceptional performance.

The sporadic Unconfined Compressive Strength (UCS) frequency peaks and troughs observed in the distribution histogram could originate from several potential sources, including binning parameters clustering divergent values during graphical translation, finite random sampling inducing random fluctuations, the presence of distinct subsurface mechanistic response subpopulations within the composite dataset combining different stabilization variables, and instrumental or methodological variability grouping values incorrectly. Further stratified analysis, parsing the measured samples by specific compositional and processing factors, would elucidate whether the deviations reflect artifactual errors or true performance bifurcation from the nuanced cement chemistry governing subgroup soil composite strengthening that warrants dedicated optimization. Isolating and addressing the root cause would allow for proper interpretation of the frequency pattern’s quality control implications.

### 3.7. UCS Values across Different Curing Times

**Figure 9:**
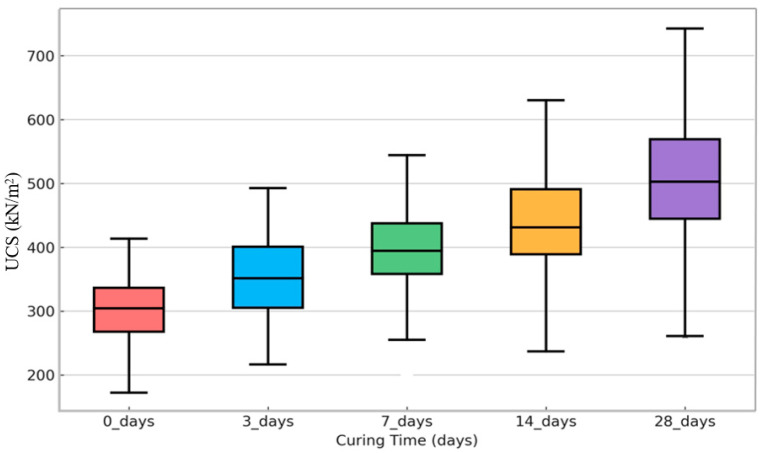
UCS values across different curing times for the fine-grained soil samples stabilized with marble powder. The bar colors represent the curing time of the mixtures: pink for 0 days, blue for 3 days, green for 7 days, yellow for 14 days, and purple for 28 days.

Figure 9 demonstrates an upward trend in unconfined compressive strength (UCS) as curing time increases from 0 to 28 days. This confirms that pozzolanic reactivity and hardening in stabilized soils progress over extended periods as more hydration products are formed [57,58,59,60]. Initially, the UCS values are pretty low without curing. This means that adding marble powder provides little immediate increase in strength before it reacts. However, significant gains are demonstrated between 0 and 7 days as the dissolution of ions accelerates. The most rapid development occurs in the 28 days, likely representing the peak formation of calcium silicate hydrates (C-S-H) and calcium aluminate hydrates from pozzolanic activity [61,62,63]. The increasing UCS is consistent with the principles of cementitious hardening and indicates a robust, sustained pozzolanic reactivity of the added marble powder. Manipulating the marble powder’s fineness, dosage, and composition could further optimize the properties. Nevertheless, the current evidence may demonstrate the consolidation potential to transform otherwise weak soils into a viable building material.

### 3.8. UCS Values across Different Molding Water Contents

**Figure 10:**
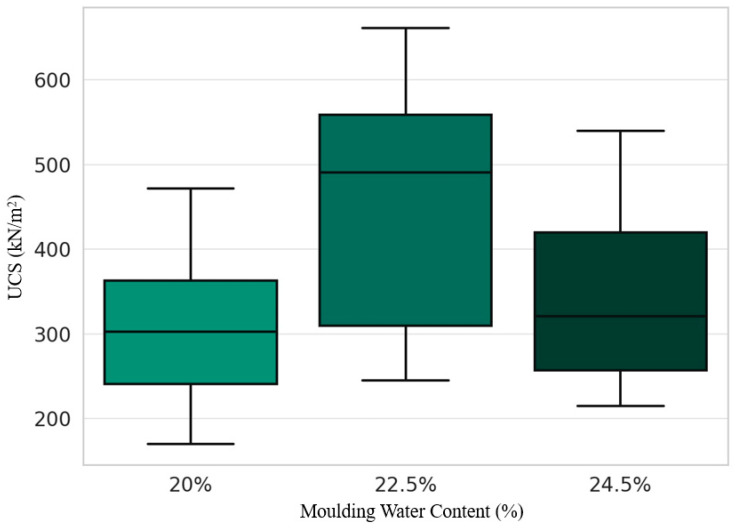
UCS values across different molding water contents for the fine-grained soil samples stabilized with marble powder. The bar green shades represent the percentage of water content added to the soil mixtures: lighter shades for 20%, medium shades for 22.5% and darker shades for 24.5%.

Figure 10 shows comparable performance over the range of molding water contents from 20% to 24.5% by dry weight. While there is some variability between samples with the same water content, the median UCS values differ only slightly. This suggests a low sensitivity of strength development to initial water content within this narrow range. According to Murthy [64], water acts as an activating agent in pozzolanic stabilization by allowing mobility and contact between soil, powder activator, and hydration products. However, excess water can also promote particle segregation and the introduction of air voids, thereby weakening the resistance. The similarity in UCS here is consistent with evidence that an optimal middle range for water content balances workability during mixing with minimized porosity, avoiding the extremes of powder segregation or incomplete pozzolanic reaction [65]. The slightly higher strength at 24.5% supports this as the ideal content, although further narrowing is needed to determine the true optimum.

Overall, the consistency suggests that water fluctuations within 4.5% of the soil dry weight do not significantly disrupt the underlying mechanisms of marble powder stabilization. This relative insensitivity and flexibility improve the practicality of field implementation where controlling exact proportions is challenging. While almost identical median performance was observed across the test range, testing below 20% and above 30% water would better delineate the lower and upper thresholds before adverse effects are seen in strength. Nevertheless, the current data confirms that there is an appropriate threshold to produce durable engineering materials.

### 3.9. UCS Values across the Different Compositions of Marble Powder

**Figure 11:**
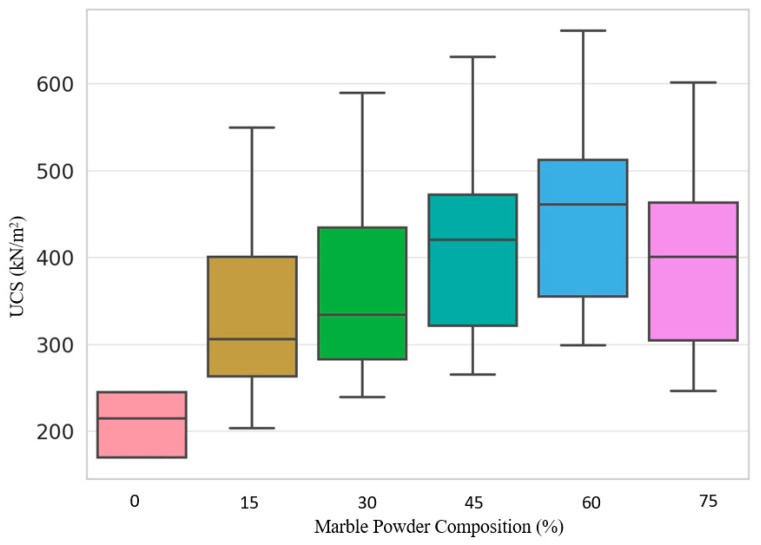
UCS values across different composition ratios for the fine-grained soil stabilized with marble powder. The bar colors represent the percentage of marble powder added to the soil mixtures: pink for 0%, brown for 15%, green for 30%, cyan for 45%, blue for 60%, and purple for 75%.

The data in Figure 11 show considerable variation in the soil and marble powder percentages tested. While UCS increased uniformly over extended curing periods, confirming ongoing pozzolanic hardening, the magnitude of strength gain varied significantly by composition. The lowest 15% marble mix exhibited the lowest UCS increase and data variability over time. This suggests that the activator cannot initiate and sustain pozzolanic reactions for consistent soil strengthening [10]. In contrast, the 60% powder dosage allowed for higher strengths and variability between samples, suggesting potential for optimization. According to Wang et al., excessive amounts of pozzolanic additives can disrupt the binding of soil fractions, introducing defects that manifest as strength inconsistencies even as mean values increase. However, too little admixture also prevents adequate cementation. An ideal balance depends on the soil mineralogy and the reactivity of the additive to allow particles to coalesce without disruption [66,67].

For the marble powder tests, the peak mean UCS probably lies at 60% additive, although precision testing is required to maximize resistance while achieving uniformity. After stabilization, reducing dispersion will also require analysis of microscopic strengthening and weakening mechanisms within the composites. Overall, the results demonstrate the feasibility and the sensitivity of using waste powders to transform weak soils into materials that meet structural requirements. However, realization depends on resolving the variability through continuity optimizations and microscale investigations to control the pozzolanic mechanisms fully. In this way, the novelty of environmentally friendly and superior alternative binders will be realized.

### 3.10. Actual vs. Predicted UCS Values

**Figure 12:**
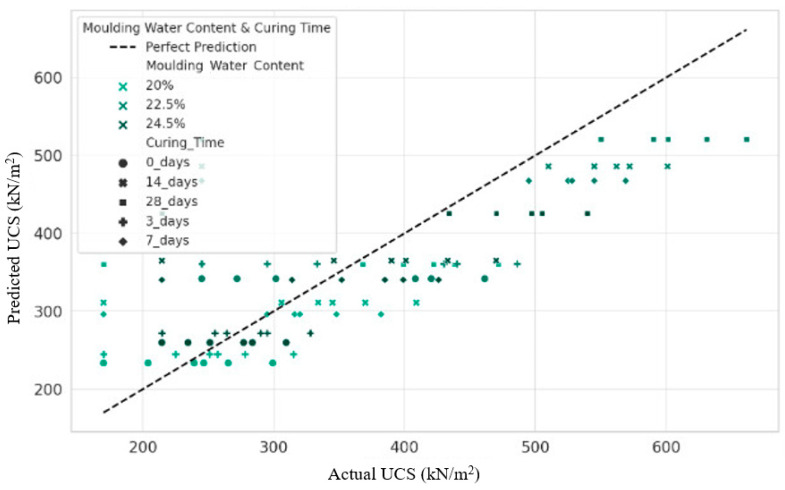
Actual vs. predicted UCS values.

The scatter plot (Figure 12) comparing experimental and model-predicted Unconfined Compressive Strength (UCS) values shows a reasonably close alignment to the parity line with minimal deviation. This indicates that the model adequately captures the dominant relationships between key input characteristics, such as curing, composition, and water content on the resulting soil composite strength. In particular, the consistency over the lower and middle UCS ranges demonstrates an accurate generalization of the core hardening phenomena and pozzolanic reactions that govern the consolidation of soil-powder mixtures. The high R-squared value quantifies this exemplary central tendency fit. However, some divergence is manifested at higher UCS extremes, suggesting that the complexity of additional variables or alternative mechanisms arising under exceptional strengthening conditions has not been correctly encoded. The model may extrapolate simplistic trends, while nuances at peaks require explicit characterization to avoid underestimation. Incorporating nonlinear feature transformations and interaction effects between composition, moisture exposure, and curing age may provide more representative embeddings at these extremes. Nevertheless, the current linear approximation proves remarkably robust.

The reliable UCS predictions, excluding outliers, confirm the model’s applicability to rapidly optimize the performance of waste-powder-amended soils once initial calibration data are available. This accelerates sustainable geomaterials’ innovation by reducing the time and trials required to formulate improved composites. However, model limitations at peak values highlight the continued need for focused physical experiments to elucidate the science that enables these heights.

The high coefficient of determination (R-squared) value of 0.954 indicates that the quartic model with fourth-degree feature transformations explains over 95.4% of the variability in the UCS measurements. The substantial gain in explanatory power through polynomial escalation is consistent with evidence that stabilized soil properties follow nonlinear relationships due to complex pozzolanic chemistry effects. Thus, simplistic linear or interaction-only assumptions may fail to capture the complex mechanisms governing consolidation. By incorporating higher degree trends, the variance of outlying forecasts is significantly increased, improving forecast accuracy. The significant reduction in RMSE to 29.82 kN/m^2^ indicates that the quartic fit significantly improves representativeness and minimizes deviations between predicted and experimental UCS values. By incorporating higher-order nonlinear feature transformations, the new model better captures the intricacies of the progressive pozzolanic reactions that govern soil–additive mixture consolidation over curing time. The significant reduction in the magnitude of the projection errors means that the quartic polynomials are tuned to the complex physicochemical underpinnings.

In contrast, more straightforward linear assumptions may fail to capture additive interactions and curing effects. This manifests as exaggerated errors when extrapolating beyond the initial data. The introduction of mathematical non-linearity allows for a more flexible fit tailored to stabilization phenomena. The metrics, therefore, quantitatively validate the merit of escalating model complexity to represent sophisticated soil gain mechanisms based on powder waste. This data-driven, chemistry-based fitting fulfills the novelty of accelerating the development of optimized and consistent renewable materials. However, avoiding overfitting requires model evaluation across diverse additive compositions and soil types. Relying solely on mathematical metrics risks masking overspecification without external validation. Computational foundations must be linked to fundamental experiments that elucidate interparticle bonding and pozzolanic reactivity to solidify the findings.

The negligible decrease of 0.022 between the high values of R-squared (0.954) and Adjusted R-squared (0.932) suggests that current polynomial modeling achieves near-optimal accuracy without substantial overfitting, as the marginal penalty indicates that any unnecessary terms would only slightly inflate the fit without further explanatory improvement. However, periodic re-examination of the adjusted R-squared as additional parameters are introduced provides quantitative guidance to prevent excessive complexity from compromising model quality and applicability in the future, even for currently well-optimized systems. In this case, the minimal deviation suggests that potential simplification gains appear modest, but the iterative application of such checks remains essential to ensure parsimony when expanding polynomial scope.

### 3.11. Normalizing Feature Importances for Model Prediction Improvement

Feature importance normalization is a technique to convert raw feature relevance scores from a model into comparable percentile contributions that sum to 1 (or 100% when expressed as a percentage) [68]. This avoids extended ranges or skewed distributions, which can lead to misleading comparisons when evaluating rankings alone. By proportionally scaling the determined impact of each variable, relative weighting and explanatory power are maintained for assessing predictive ability [69]. Meanwhile, the adjusted comparison scale from 0 to 100% better highlights both overwhelming and subtle differences between contributors that may be missed without normalization. For example, if trait A originally scored 0.95 and B scored 0.05, normalization as 95% and 5% better highlights the dominant role of A over B. The same absolute difference of 0.9 and 0.1 seems less dramatic and more difficult to disentangle comparative influence. Expanding to more variables and smaller gaps maintains this interpretable scale. In addition, the total contribution percentages allow for hierarchical structuring of features into different levels of importance categories—from primary parameters that each explain more than 30% to ancillary parameters that contribute less than 5%. This stratification provides actionable insights for directing resources.

**Figure 13:**
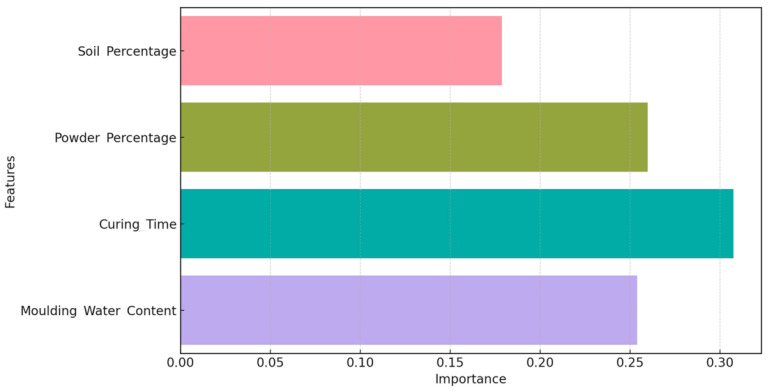
Normalizing feature importances for model prediction improvement. The bar colors represent the percentage of parameters importance: pink for soil, green for marble, cyan for curing time, and purple for molding water content.

As seen in Figure 13, curing time stands out as the most influential parameter impacting UCS, with over 32% weighting. This aligns with Wang et al., who demonstrated enduring strength development in stabilized soils corresponding directly to progressive pozzolanic activity over months [70]. The second and third-ranked features—molding water content and marble powder percentage—exhibit comparable contributions of around 26% each. This corroborates evidence that water proportions suitable for cementation reactions and sufficient activator contents are vital for consolidation [1]. However, the magnitude gap compared to curing age signifies that these are necessary but not independently adequate conditions.

The fact that curing time is the most critical factor in UCS development is consistent with expectations for Portland cement-stabilized soils. Portland cement requires sufficient hydration and pozzolanic reaction time to impart strength gains in composites. Because Portland cement bonding involves initial dissolution and precipitation reactions between cement grains, water, and soil minerals over hours, followed by further conversion of hydration products and additional solubilization of silica/alumina reactants from soils over weeks, strength enhancement is inherently time-dependent. Therefore, the overriding importance of curing time for UCS enhancement is consistent with the multifaceted, progressive chemistry involved in Portland cement stabilization. Sufficient aging catalyzes pozzolanic activity between soil particles, aggregate minerals, and cement degradation products. This matures cross-linked calcium silicate hydrate binders responsible for hardening the soil composite.

In contrast, non-cementitious alternative stabilizers may act through more rapid physicochemical changes, such as ion exchange, to gain strength shortly after mixing. However, the 32% characteristic importance for Portland cement is an expected result based on the need for sustained cementitious reactions over months before optimal setting occurs. Overall, this is consistent with the time-dependent setting mechanism of Portland cement.

Interestingly, despite being the predominant composite fraction, the parent soil component generates 18% importance. This implies that inherent soil properties play a secondary role if essential index criteria are satisfied [71]. Instead, the waste powder additive and time for pozzolanic hardening dominate control. The feature weights, therefore, quantitatively distinguish the hierarchy of factors regulating UCS outcomes. Curing duration emerges as the principle lever that compounds activator and water optimizations to transform weak soils into hardened composites. While the model results indicate that the percentage of parent soil contributes the least of the variables tested, this does not imply that the soil properties are insignificant overall. Instead, it suggests that adequate pozzolanic reactions mitigated the variability of the specific soil used within the compositional range evaluated, allowing other factors to become more rate-limiting. However, increasing the diversity of soils, especially across extremes of plasticity, organic content, pH, etc., would likely change this lower relevance ranking. High-plasticity clay soils may consume more cement before reaching maximum stabilization. Organic matter can inhibit cement bonding. Acidic sulfates can corrode cement by chemical attack. The importance of soil specifics would thus become more apparent. In addition, different soils have different availability of reactive minerals. Clay and siliceous soils allow abundant cementitious gel formation and bonding, while quartz sands and calcareous soils provide limited reactants. Therefore, the soil’s reactive potential would significantly affect the results.

In terms of novelty, applying automated model feature screening on composition variables and aging timelines for pozzolana-treated soils provides a rapid assessment toolkit. While laboratory testing explores limited combinations, intelligent algorithms can interpolate expected patterns to guide efficient optimizations. This brings enhanced precision and performance towards recycling waste into sustainable geo-materials. However, model reliability depends on initial representative data spanning influential feature ranges. Insights should thus supplement rather than replace carefully designed physical experiments that elucidate controlling chemistry and microstructure behind statistical trends.

Although extensive testing was conducted, the soil samples were from a single site, and the marble powder was from a specific industrial source. Expanding the diversity of soils and additive powders could improve the generalization of trends across soil types and compositions. While nonlinear trends were incorporated into subsequent quartic regression modeling, interactions between influential features such as compositional ratios, water content, and curing age were not fully captured. Developing more complex models that account for factorial effects could better represent interactions and optimize predictions. The models also exclude physicochemical factors that drive the observed strength patterns: as is typical of laboratory studies, practical considerations for field mixing, quality control of proportions, and variability in loading and environmental exposures were not addressed. Further work is needed to translate and validate the idealized experimental conditions to real-world construction applications. Monitoring of demonstration projects would indicate robustness.

## 4. Conclusions

This study achieved immense progress in demonstrating the feasibility of repurposing waste marble powders to strengthen fine-grained soils sustainably. The considerable strength improvements and favorable modeling metrics quantify the potential to transform waste materials into robust infrastructure composites. By methodically addressing these future directions, the promising initial foundation from this work can mature into a powerful and optimized soil-strengthening solution. The quantitative framework and multi-disciplinary techniques pioneered here will be the springboard for reliable infrastructure adoption. Realizing the full capability and consistent performance vital for extensive adoption will require systematic refinement across several dimensions, including:

1. The addition of marble powder significantly improved the geotechnical properties of the fine-grained clayey soil. The LL decreased from 62.2% to 34.29%, the PL decreased from 29.6% to 13.88%, and the PI reduced from 32.6% to 18.41%. The MDD increased from 1.65 to 2.441 g/cc, and the UCS increased from 170 to 661 kN/m^2^ after 28 days of curing with the optimum marble powder content of 60%, then at 75% powder, which led to a decrease in the UCS, with values dropping to 601 kN/m^2^.

2. The optimum marble powder content for maximizing the soil’s engineering properties was around 60% by dry weight. The CBR increased from 10.43% to 22.94% for unsoaked and 4.68% to 12.46% for soaked conditions with 60% marble powder. The ITS increased from 100 to 208 kN/m^2^ with 60–75% marble powder.

3. The curing time was crucial in developing UCS for Exploratory Data Analysis (EDA) techniques. The UCS values remained low, around 237 kN/m^2^, at 0 days of curing but increased significantly to 546 kN/m^2^ after 28 days. Quartic regression modeling achieved an R^2^ value of 0.954 and RMSE of 29.82 kN/m^2^ in predicting the UCS response of stabilized soil based on features such as curing duration, marble powder percentage, and molding water content.

4. Expand research to conduct more laboratory tests and field trials to validate the robustness and applicability of the proposed predictive model under different environmental and loading conditions, such as temperature, humidity, freeze–thaw cycles, and dynamic loads.

## Figures and Tables

**Table 1:** Test protocol.

S/N	Test Class		w (%)	Tests	Curing Period (Days)
	Natural soil (%)	Marble powder (%)			
1	100	0	20, 20.5, 24.5	UCS	0, 3, 7, 14, 28
2	85	15			
3	70	30			
4	55	45			
5	40	60			
6	25	75			

**Table 2:** The basic properties of the studied soil.

Basic Properties		Amount
**Specific Gravity, %**		2.73
**Atterberg Limits**	Liquid Limit, %	62.2
	Plastic Limit,%	29.6
	Plasticity Index, %	32.6
**Finer Component**	% Passed NO. 200 Mesh, %	71.6
**USCS Classification**	CH	-
**Compaction Parameter**	Optimum Moisture Content, %	22.5
	Maximum Dry Density, Mg/m^3^	1.60
**UCS, kN/m^2^**	-	245
**Initial Void Ratio (e_o_)**	-	0.665
**Color**	-	Dark Beige
**PH**	-	5.51
**Dominate soil mineral**	-	Kaolinite

**Table 3:** Chemical composition of the marble powder.

Composition	CaO	SiO_2_	P_2_O_5_	Fe_2_O_3_	Al_2_O_3_	MnO	Na_2_O	K_2_O	MgO	LOI	Total
**Values (%)**	56.33	0.28	0.01	0.37	0.07	0.01	0.06	0.02	0.65	42.27	100.07

**Table 4:** Unconfined Compressive Strength (UCS) for 0-, 3-, 7-, 14- and 28-days curing time at various molding water content.

Soil/Powder Composition	UCS (kN/m^2^) for 0 Day	UCS (kN/m^2^) for 3 Days	UCS (kN/m^2^) for 7 Days	UCS (kN/m^2^) for 14 Days	UCS (kN/m^2^) for 28 Days
20% molding water content					
100%soil/0%powder	170	170	170	170	170
85%soil/15%powder	204	225	295	306	368
70%soil/30%powder	239	251	316	334	399
55%soil/45%powder	265	278	348	370	438
40%soil/60%powder	299	315	382	409	472
25%soil/75%powder	247	257	320	345	422
22.5% molding water content					
100%soil/0%powder	245	245	245	245	245
85%soil/15%powder	272	295	495	510	550
70%soil/30%powder	302	333	525	545	590
55%soil/45%powder	420	440	545	572	631
40%soil/60%powder	461	486	569	601	661
25%soil/75%powder	408	430	528	562	601
24.5% molding water content					
100%soil/0%powder	215	215	215	215	215
85%soil/15%powder	235	255	314	346	434
70%soil/30%powder	251	264	352	390	470
55%soil/45%powder	284	295	399	433	505
40%soil/60%powder	310	328	426	470	540
25%soil/75%powder	277	290	385	401	497

**Table 5:** Basic statistics of Unconfined Compressive Strength (UCS) for 0-, 3-, 7-, 14- and 28-day curing time at various molding water contents.

Curing Time	Mean UCS (kN/m^2^)	Standard Deviation (kN/m^2^)	95% CI Lower Bound (kN/m^2^)	95% CI Upper Bound (kN/m^2^)
20% molding water content				
0 day	237.3871	45.49191	189.6463	285.128
3 days	249.3333	49.15554	197.7478	300.9189
7 days	305.1667	72.73078	228.8404	381.4929
14 days	322.3333	82.34723	235.9152	408.7515
28 days	378.1454	107.8354	264.979	491.3117
22.5% molding water content				
0 day	351.4384	89.61126	257.3971	445.4796
3 days	371.5	94.40498	272.4281	470.5719
7 days	484.5	119.8361	358.7397	610.2603
14 days	505.8333	131.282	368.0613	643.6054
28 days	546.4111	152.3853	386.4925	706.3297
24.5% molding water content				
0 day	261.7992863	34.85796559	225.2180949	298.3804778
3 days	274.4291991	38.99795432	233.503356	315.3550423
7 days	348.4291991	76.19327185	268.4692641	428.3891342
14 days	375.7625325	89.29012196	282.0582962	469.4667688
28 days	443.3529503	117.530932	320.0118014	566.6940991

## Data Availability

Some or all data, models, or codes that support the findings of this study are available from the corresponding author upon reasonable request.
